# Meridian study on the response current affected by electrical pulse and acupuncture

**DOI:** 10.1186/s11671-020-03373-2

**Published:** 2020-07-10

**Authors:** Yu-Chiang Hung, Wen-Chung Chen, Ting-Chang Chang, Hao-Xuan Zheng, Yan-Wen Liu, Yung-Fang Tan, Shih-Kai Lin, Ying-Hsin Lu, Wen-Long Hu, Tsung-Ming Tsai

**Affiliations:** 1 Department of Chinese Medicine, Kaohsiung Chang Gung Memorial Hospital, 123 Dapi Road, Kaohsiung, 83301 Taiwan; 2 School of Chinese medicine, Chang Gung University College of Medicine, 259, Wenhua 1st Rd., Guishan Dist., Taoyuan, 33302 Taiwan; 3 Department of Materials and Optoelectronic Science, National Sun Yat-Sen University, 70 Lienhai Rd, Kaohsiung, 80424 Taiwan; 4 Department of Physics, National Sun Yat-Sen University, 70 Lien-hai Road, Kaohsiung, 80424 Taiwan; 5 The Center of Crystal Research, National Sun Yat-Sen University, Kaohsiung, 804 Taiwan; 6 Institute of Electronics Engineering, National Tsing Hua University, Hsinchu, 30013 Taiwan; 7 College of Nursing, Fooyin University, 151, Jinxue Rd, Kaohsiung, 83102 Taiwan; 8 Kaohsiung Medical College of Medicine, 100, Shiquan 1st Rd., Kaohsiung, 80708 Taiwan

**Keywords:** Acupuncture, Chinese medicine, Electrical pulse, Meridian theory, Ion current

## Abstract

Acupuncture and its meridians are important components of traditional Chinese medicine, and numerous opinions have been previously expressed regarding these meridians. This study aims to explore the phenomenon of meridians from the perspective of electronic physics by studying these meridians for the response current affected by electrical pulse and acupuncture. In this study, acupuncture which applies an electrical pulse was used to research the physical properties of the meridians. Different kinds of pulses were applied to the human body to realize abnormal electrical signals. Comparing these electrical measurement results with the isothermal transient ionic current (ITIC) theory, we found that the transmission of meridian messages may be related to ion conduction. The movement of ions induced by acupuncture and electrical stimulation can lead to drift and diffusion currents through the meridians. The ionic conduction of meridian hypothesis is proved in that the substances delivered by meridians are in fact ions.

## **Introduction**

Acupuncture has been used for more than three thousand years. Now it is not only known as an important treatment instrument for musculoskeletal illness, but is also widely used to treat internal-medical diseases [1–4]. Though acupuncture is widely used for healing the disease in human body, better clarity is needed of the composition of meridians and their fundamental theory [5–7].

Meridian theory is a core component and foundation of acupuncture and *tui-na* massage. There are many different kinds of hypothesis on the nature of meridians [8]. They can be divided into four theoretical parts: those of nerve conduction theory [9, 10], body fluid circulation theory [11, 12], fascia and connective tissue structure doctrine [13, 14], and biological field (or energy) doctrine [15, 16]. Each hypothesis interprets the meridian essence of Chinese medicine from a different perspective, and these different hypotheses try to explore the essence of the meridian from individual reasonable points of view. However, each hypothesis can only explain part of the meridian essence and cannot provide a comprehensive interpretation of the meridian system. This study aims to explore the phenomenon of meridians from the perspective of electronic physics by comparing different kinds of pulses to explain an abnormal current, proving that the movement substance is ions.

Electroacupuncture involves the insertion of needles into acupoints and introducing an electrical current through that needle, thus combining electricity and the needle to enhance the stimulation and the effects of acupuncture. When choosing two or more meridians to use electroacupuncture, the acupoint and meridian act like electrodes and a current channel, such that the current passes through the meridians. By applying direct and alternating pulses into the *hegu* and *quchi* points in this work, the oscilloscope signal and responding current can be measured. This is very similar to the ITIC theory. For ions, it is very small substance moving to form the current [17–20]. In addition, many nanomaterials and devices used this theory to explain their model [21–24]. It is important to realize the transport mechanism in the ITIC theory. Moreover, by introducing the ion current model and comparing the difference in response current from direct and alternating pulses, the physical characteristics of meridians can be understood more clearly.

## **Experimental Methods**

### **Participant Selection**

This clinical trial was approved by the Institutional Review Board of Chang Gung Medical Foundation (IRB permit no. 201800392A3). Written informed consent was obtained from all participants before enrollment. After certain initial assessments, 30 volunteers who met the inclusion criteria mentioned below were recruited; they included 15 men and 15 women, aged between 20 and 30 years. They were all well informed about this project and signed the consent before the experiment. We chose healthy volunteers as subjects in this study and therefore did not target any particular acupoint. The positions were instead chosen for their common use and convenient location in order to observe the phenomenon of ion transport in meridians.

### **Inclusion and Exclusion Criteria**

According to a prospective survey, despite its benefits, acupuncture can also lead to some side effects [25]. As an invasive treatment, it may sometimes induce either local or systemic adverse reactions [25–27]. Moreover, a systemic review shows that life-threatening events may also develop, albeit rarely. As a result, the exclusion criteria were very stringent. Bleeding and hematoma are the most common adverse reactions. Volunteers with bleeding tendencies (platelet counts less than 20,000 and/or thrombocytopenic purpura) were excluded. Volunteers with chronic medical conditions who were prescribed anti-coagulants were also excluded. In addition, pregnant women and volunteers with pacemakers were excluded.

### **Acupuncture Needles**

The acupuncture needles used in this experiment were produced under the same conditions, in the same factory (Dong Bang Acupuncture Inc.), and on the same day, to minimize experimental error.

### **Experiment Process**

**Fig. 1:**
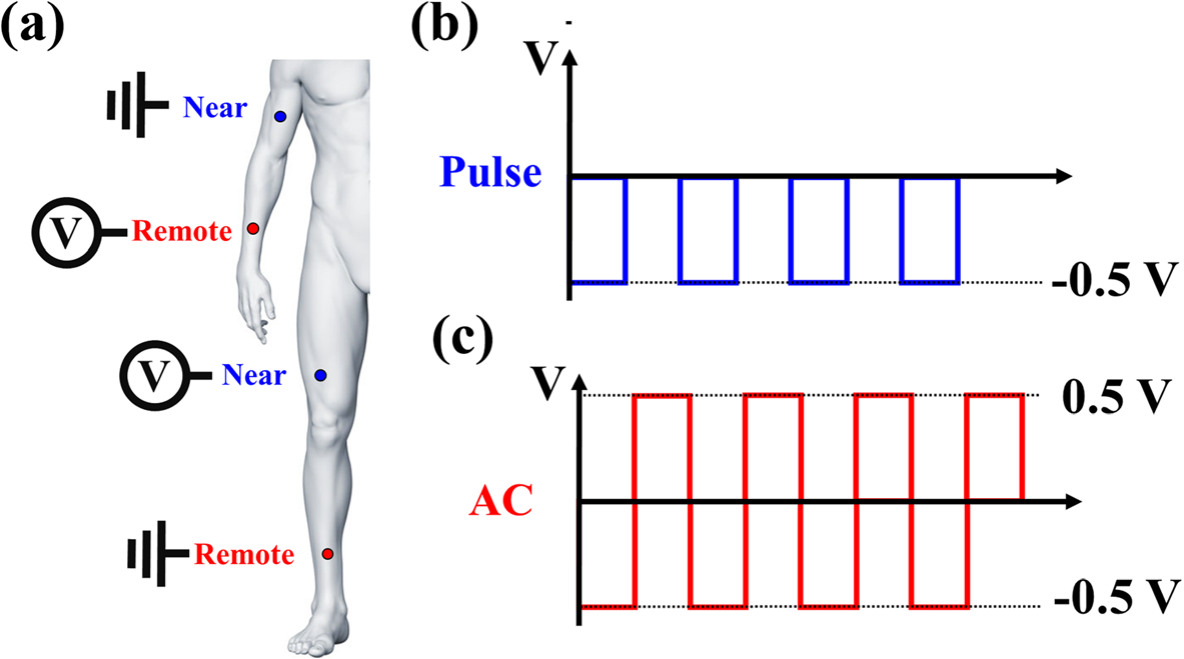
**a** The schematic of measurement mode. Applying the voltage at the remote point is defined as remote electroacupuncture, and another one, applying the voltage at the near point is defined as near electroacupuncture. The schematic of pulse types of **b** pulse and **c** AC

In this experiment, the semiconductor measurement analyzer Agilent B1500A is used to input the human body meridian waveforms, and the flow directions of electrical signals are defined as remote electroacupuncture and near electroacupuncture, respectively, as shown in Fig. 1a. Remote electroacupuncture means that the electrical signals go through from limbs to body, and near electroacupuncture means that the electrical signals go through from body to limbs. The Chinese medicine treatment of electroacupuncture usually uses a square waveform. This is the reason we decided to use square waveforms, instead of triangle waveforms. In addition, when applying voltage, two different waveforms are given by alternating current (AC) and pulse, as shown in Fig. 1b. AC is a continuous square wave going back and forth between 0.5 and − 0.5 V, and pulse is a continuous square wave starting from 0 to 0.5 V. Further, the experiment uses AC and pulses of four frequencies (2, 4, 6, and 8 Hz). All data evaluations in this study were repeated thrice, which are presented as mean ± standard deviation.

## **Result and Discussion**

**Fig. 2:**
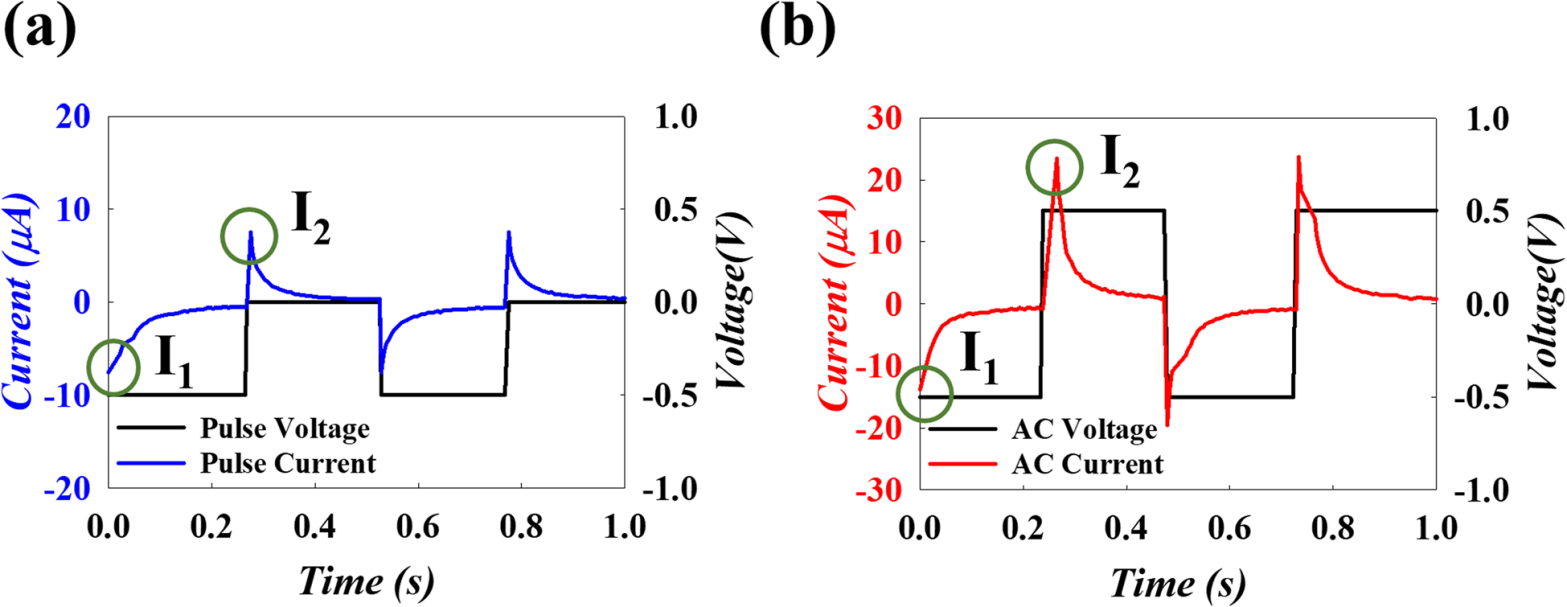
The measurement of time versus voltage and current for **a** pulse and **b** AC, with *I*_1_ and *I*_2_ indicated in both

First, we chose the LI meridian with remote and near electroacupuncture between *hegu* and *quchi* points to start the experiment and measured the responding current. For AC, as the voltage was 0.5 V, the response current was 13 μA, and the current was 26 μA at 0.5 V, as shown in Fig. 2a. In contrast, the current of the pulse waveform also read 10 μA when the voltage was at 0.5 V. However, there was an abnormal reverse current of about 8 μA at voltage of 0 V, as can be seen in Fig. 2b.

**Fig. 3:**
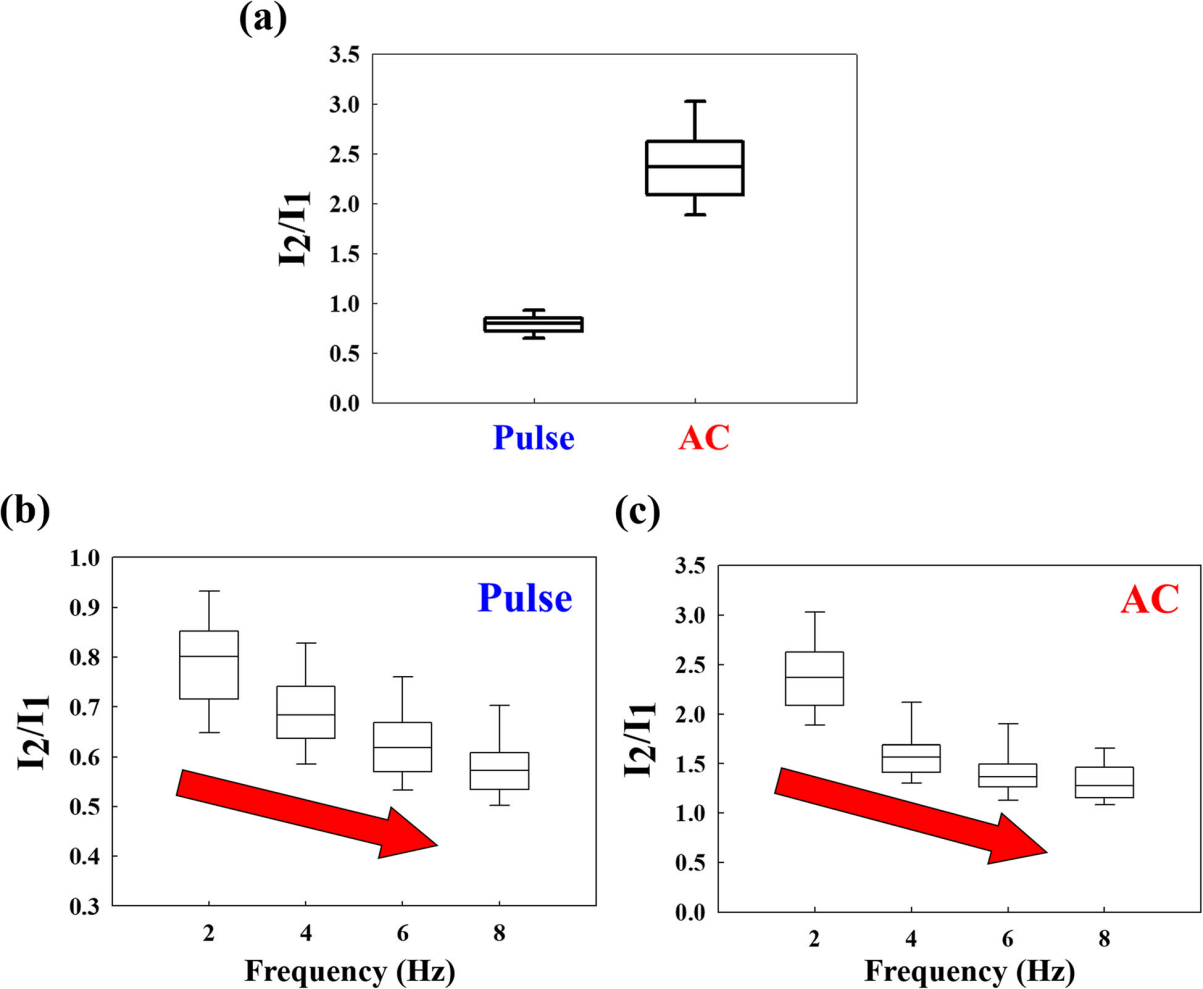
**a** Comparison of *I*_2_/*I*_1_ ratio for pulse and AC. The ratio in AC is larger than that in pulse. *I*_2_/*I*_1_ ratio for **b** pulse and **c** AC decrease with increase in frequency

In order to analyze this abnormal current, the initial current value was defined as *I*_1_, and the abnormal reverse current was defined as *I*_2_. We divide *I*_2_ by *I*_1_ to find the difference between pulse and AC. The pulse ratio was close to 1, which indicates that the current is negative at negative voltage, but will reverse back at the same amount without any voltage applied. However, in the AC ratio, the value was close to 2. This indicates a forward current at a negative voltage, but when reverse voltage is applied, the current will not only flow back but also increase with the response current. Therefore, the AC ratio becomes larger than the pulse ratio, as shown in Fig. 3a. Similarly, we change different frequencies and plot the ratio of *I*_2_/*I*_1_ against the frequency. In the pulse experiments, we found that the higher frequency showed a smaller ratio because there was not enough time for the compensation current in the higher frequency (shorter period) pulse, as shown in Fig. 3b. In addition, AC experiments with different frequencies demonstrate the same trend, as shown in Fig. 3c. The ratio decreased as the frequency increased in both pulse and AC experiments.

The *I*-*V* curve was used to analyze the mechanism inside the meridians of the human body. In the beginning, the current increases sharply and gradually decreases and saturates after exceeding the peak maximum value. We found that this was similar to the isothermal transient ionic current (ITIC) mechanism [28–33]. In the ITIC theory, the anions and cations will move by electrical field to the two sides of the channel, resulting in the increase in current [34]. When the ions accumulate at the two sides, the current will gradually decrease because of the barrier from the space charge. Therefore, the way of conducting electricity in the human body is mainly through ions.

**Fig. 4:**
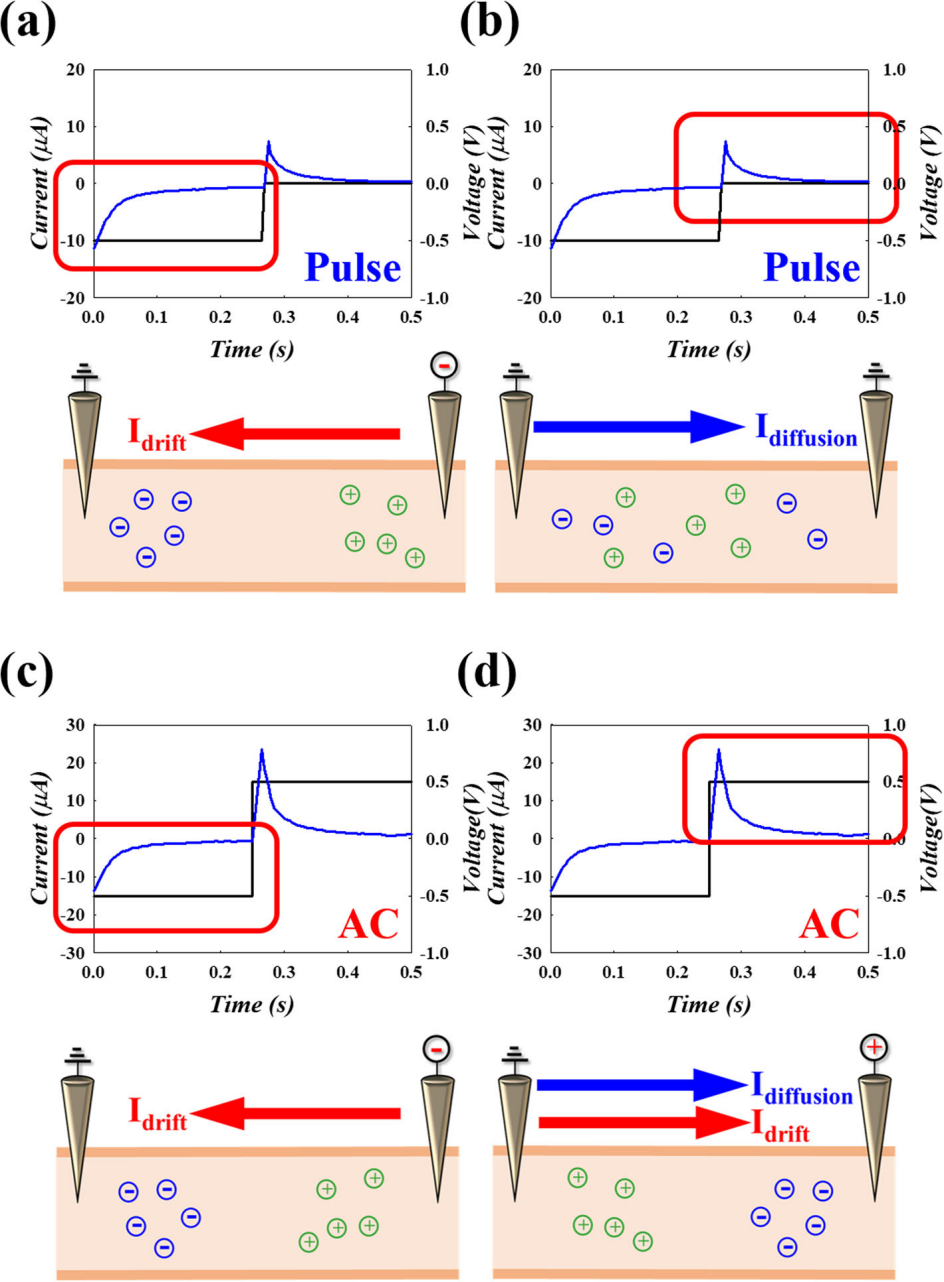
The model for explaining the phenomenon of the pulse electric feature **a** drift current caused by voltage and **b** diffusion current caused by concentration gradient. For AC electric feature **c** drift current caused by voltage; **d** reverse voltage generate the larger current than diffusion current of pulse

From the ITIC theory, the electrical signal transference in meridians is very similar to the ion current. As a result, following measurement results and the ITIC theory, for applied voltage of 0.5 V at initial, the ions will move by the electric field, leading to drift current, and will accumulate at the two sites, as shown in Fig. 4a. However, when the voltage was not applied, the abnormal current was read by the diffusion current of the different concentration of ions. Therefore, the current direction was opposite to that of the drift current, as shown in Fig. 4b. Similarly, in AC pulse conditions, 0.5 V was applied, leading to the drift current in Fig. 4c. However, when the voltage changed from 0.5 to − 0.5 V, both diffusion current and drift current with the opposite electric field were formed, leading to a current twice than measured at initial, as shown in Fig. 4d.

According to this result, the same experiments were carried out in 12 meridians. In Table 1, the ratio of *I*_2_/*I*_1_ all show the same result. Pulse had an abnormal response current, and the ratio was close to 1, while the ratio in AC was about 2.

**Table 1:** Average and standard deviation of *I*_2_/*I*_1_ ratio to AC and pulse for 12 meridians

Meridians	AC	Pulse
*Yangming* large intestine meridian in hand (LI)	1.57 ± 0.53	0.51 ± 0.18
*Taiyang* small intestine meridian in hand (SI)	1.66 ± 0.42	0.54 ± 0.14
*Shaoyang Sanjiao* meridian in hand (SJ)	1.57 ± 0.33	0.64 ± 0.11
*Shaoyin* heart meridian in hand (HT)	1.25 ± 0.38	0.73 ± 0.63
*Taiyin* lung meridian in hand (LU)	1.59 ± 0.30	0.64 ± 0.12
*Jueyin* pericardium meridian in hand (PC)	1.60 ± 0.32	0.64 ± 0.15
*Taiyang* bladder meridian in foot (BL)	1.76 ± 0.46	0.77 ± 0.32
*Shaoyang* gallbladder meridian in foot (GB)	1.55 ± 0.42	0.64 ± 0.10
*Yangming* stomach meridian in foot (ST)	1.52 ± 0.46	0.53 ± 0.14
*Shaoyin* kidney meridian in foot (KI)	1.69 ± 0.46	0.63 ± 0.13
*Jueyin* liver meridian in foot (LR)	1.59 ± 0.38	0.50 ± 0.16
*Taiyin* spleen meridian in foot (SP)	1.38 ± 0.50	0.58 ± 0.15

Our findings are similar to the body fluid circulation theory. Acupuncture on the meridian acupoint can induce ion passive diffusion. In body fluid circulation theory, the meridians consist of ions and neurotransmitters, because human tissue is a complex electrolyte electrical conductor composed of moisture, inorganic salts, and charged biocolloids. When a pulse is applied to the human body, the ions will move directionally, eliminating the polarization of the cell membrane. Consequently, the ion concentration and distribution shows significant variety, affecting human tissue function. It is also the basic electrophysiological element.

One limitation of this study is that diseases may negatively affect the signal transmission of the meridian. We enrolled 30 volunteers, but not patients, to participate in this research. Electronic signal response in acupuncture meridians of patients suffering a specific disease require future study.

## **Conclusion**

This study used different kinds of pulse (pulse and AC) to realize abnormal response current by the reverse voltage. This abnormal current can be explained via the ITIC theory, demonstrating that the movement of ions leads to the drift and diffusion currents. Therefore, the ions can be confirmed to be the substance transferred by the meridians.

## Data Availability

All data are fully available without restriction.

## Abbreviations

ITIC: Isothermal transient ionic current
AC: Alternating current
LI: *Yangming* large intestine meridian in hand
SI: *Taiyang* small intestine meridian in hand
SJ: *Shaoyang Sanjiao* meridian in hand
HT: *Shaoyin* heart meridian in hand
LU: *Taiyin* lung meridian in hand
PC: *Jueyin* pericardium meridian in hand
BL: *Taiyang* bladder meridian in foot
GB: *Shaoyang* gallbladder meridian in foot
ST: *Yangming* stomach meridian in foot
KI: *Shaoyin* kidney meridian in foot
LR: *Jueyin* liver meridian in foot
SP: *Taiyin* spleen meridian in foot
